# Editorial: MALDI-TOF MS in microbiological diagnostics: future applications beyond identification

**DOI:** 10.3389/fmicb.2023.1204452

**Published:** 2023-04-25

**Authors:** Karsten Becker, Antonella Lupetti

**Affiliations:** ^1^Friedrich Loeffler-Institute of Medical Microbiology, University Medicine Greifswald, Greifswald, Germany; ^2^Dipartimento di Ricerca Traslazionale e delle Nuove Tecnologie in Medicina e Chirurgia, Università di Pisa, Pisa, Italy

**Keywords:** antimicrobial resistance, diagnostics, mass spectrometry, MALDI-TOF, susceptibility testing, nano LC-MS/MS

Following a Research Topic on matrix-assisted laser desorption/ionization time-of-flight (MALDI-TOF) mass spectrometry (MS) application for susceptibility testing of microorganisms (Becker and Schubert, 2021), three main reasons prompted us to initiate another one on future applications of MALDI-TOF and other MS approaches beyond identification. Firstly, the silent pandemic of multi-drug resistant organisms (MDROs) as designated by the WHO undoubtedly needs more attention. While the development of new antibiotics is receiving due attention, the development of feasible tests for rapid and reliable detection of antimicrobial resistance (AMR) is still passing under the radar of public attention threshold (van Belkum et al., 2013, 2019; Idelevich and Becker, 2019). Beyond the individual treatment, antibiotics could be considered “social” pharmaceuticals as they may also have general effects. Similarly, diagnostic tests used for microbial identification and antibiotic susceptibility testing (AST) deserve to be considered “social” diagnostics with comparable attention and support as antibiotics, since incorrect or late test results can negatively influence both the antibiotic treatment for the patient and MDRO prevention measures, thus leading to avoidable MDRO transmission and increase selection pressure through unnecessarily broad therapy.

The second reason regards sustainability and cost efficiency. MALDI-TOF MS applications beyond identification may ideally expand the application possibilities of a diagnostic device already placed in many routine laboratories (Clark et al., 2013; Schubert and Kostrzewa, 2017; Welker et al., 2019). Not one method alone can meet all of today's requirements for a full, rapid, robust, little personnel-intensive, cost-effective, high-throughput and routine-ready AMR diagnostic approach with excellent sensitivity and specificity. MALDI-TOF MS has the advantage to represent a phenotypic approach, allowing resistance mechanism-independent assays, as known from classical growth-based AST. Besides specific resistance mechanism-based MALDI-TOF MS assays, e.g. addressing enzyme-caused alterations of an antibiotic (Sparbier et al., 2012), universal approaches have been already reported, as the direct-on-target microdroplet growth assay (DOT-MGA) (Idelevich et al., 2018). Further MALDI-TOF MS advantages include rapidity and random access opportunity.

Thirdly, to consider novel MALDI-TOF MS developments not related to identification and AST. The current main efforts to expand the application profile of MALDI-TOF MS beyond identification target its use for AST as reflected by most of the original articles published in this Research Topic. However, they do not refer to the further development and optimization of the technical procedures for AST itself, but to support this process e.g., by machine learning (ML) software tools.

Nowadays, there is an increased effort to take advantage of artificial intelligence (AI) algorithms to analyze the patterns of MALDI-TOF MS peaks for microbial identification and AST (Weis et al., 2020). By the example of the foodborne pathogens *Campylobacter coli* and *C. jejuni*, Feucherolles et al. describe a ML prediction approach as an AMR screening tool for seven antimicrobial resistances. The aim of diagnostic tests is achieving both high sensitivity and specificity. This study shows the potential of this approach, but also the unsolved hurdles to reach the necessary balance between sensitivity and specificity. Here, high sensitivity was chosen as the most important parameter to adjust the threshold score during the tuning part, which led to specificity problems regarding some of the antibiotics tested. The authors concluded that threshold adjustment is vital while elaborating ML pipeline for routine use based on MALDI-TOF mass spectra. Wang H-Y. et al. used ML approaches to construct a prediction model for rapid detection of ciprofloxacin-resistant *Klebsiella pneumoniae* strains based on identified significant biomarkers. However, as with similar ML approaches, they realized that those models cannot be generalized to other microbial species and antibiotics. This limitation has been addressed by Zhang et al. on serial antibiotic resistances prediction. They generated a multi-label prediction model for clindamycin and oxacillin susceptibilities in *Staphylococcus aureus* based on MALDI-TOF MS data. In this context, multi-label learning targets the challenge where each case is represented by a single instance while simultaneously related with a set of labels (Zhang and Zhou, 2014). Wang C. et al. reported a deep learning-based algorithm on a convolutional neural network (CNN) for that a benchmarking study has recently shown that it is able to outperform traditional machine learning methods (Mortier et al., 2021). Here, utilizing the complete information of MALDI-TOF mass spectra for detecting *Enterococcus faecium*, a CNN model to rapidly and accurately predict clinical vancomycin-resistant *E. faecium* (VREfm) was introduced.

Over the past decade, numerous efforts have been made for direct identification and AST of microorganisms in positive blood cultures (BCs). Verroken et al. evaluated the performances of a commercially available system designed to isolate and concentrate microbial cells directly from a positive BC bottle, the so-called “liquid colony,” as equivalent of a overnight subcultured colony for identification by MALDI-TOF MS and other purposes.

The polymyxin colistin, a polycationic peptide antimicrobial, is still considered as last line of defense against carbapenemase-producers. Determination of resistance by MS-based assessment of the negatively charged lipopolysaccharide component lipid A as cellular target of the polymyxins is favored by switching the MALDI-TOF-MS device into the negative-ion mode (Dortet et al., 2018). Jeannot et al. evaluated the MALDIxin test for the detection of colistin-resistant *Pseudomonas aeruginosa* clinical strains. All colistin-susceptible and most of the resistant strains were detected in <1 h; the remaining resistant strains were detected after 4-h colistin pre-exposure. However, this procedure needs a spectrometer equipped with a respective switching modality and it creates more effort, e.g., by additional calibration steps, because the positive-ion mode is used for routine diagnostic purposes in clinical microbiology. For this reason, Foglietta et al. developed a MALDI-TOF MS test in positive-ion mode for rapid detection of colistin-resistant *K. pneumoniae* after a 3-h incubation reaching total agreement with the phenotypic reference method.

Gato et al. evaluated the performance of MALDI-TOF MS for rapid detection of carbapenemase activity in *Enterobacterales*, with a standardized procedure with online software for data analysis using carbapenemase-producers and controls in a multicenter study, followed by a 2-month period clinical evaluation. The accuracy ranged from 83.3 to 100% among the eight international centers. Shaidullina et al. evaluated disks containing ertapenem as source of substrate in MALDI-TOF MS-based assay for detection of carbapenemase-producing *Enterobacterales* and found a sensitive, specific and cost-effective alternative. Lu et al. introduced a nanoscale liquid chromatography coupled to tandem MS (nano LC-MS/MS) workflow to detect enterobacterial carbapenemases with a screening method based on peptide groups (14 peptides with 100% specificity, nine peptides with 95–100% sensitivity).

Olson et al. used MALDI-TOF MS to genotype *Moraxella bovis* strains based on two biomarker models to classify strains according to genotype with an overall accuracy of 85.8–100%.

In summary, the main conclusion that can be drawn from this Research Topic is that the development of non-identification MALDI-TOF MS applications is worth continuing. The overriding advantage is its special potential to obtain a lot of information from one single protein spectrum analysis, i.e., species identification, AST results, data of the genetic diversity and, possibly, further information e.g., on virulence factors.

## Author contributions

KB and AL managed the Research Topic MALDI-TOF MS in Microbiological Diagnostics: Future Applications Beyond Identification as topic editors. KB and AL wrote the manuscript and both authors reviewed and edited the manuscript.
